# Preparation and Modification Technology Analysis of Ionic Polymer-Metal Composites (IPMCs)

**DOI:** 10.3390/ijms23073522

**Published:** 2022-03-24

**Authors:** Chendong He, Yunqing Gu, Junjun Zhang, Longbiao Ma, Muhan Yan, Jiegang Mou, Yun Ren

**Affiliations:** 1 College of Metrology & Measurement Engineering, China Jiliang University, Hangzhou 310018, China; p20020854018@cjlu.edu.cn (C.H.); p20020854106@cjlu.edu.cn (J.Z.); s20020804040@cjlu.edu.cn (L.M.); s20020804068@cjlu.edu.cn (M.Y.); 2 Zhejiang Engineering Research Center of Fluid Equipment & Measurement and Control Technology, Hangzhou 310018, China; 3 Zhijiang College, Zhejiang University of Technology, Shaoxing 312030, China; renyun-ry@hotmail.com

**Keywords:** IPMC, smart materials, driving mechanism, preparation technology, modification research

## Abstract

As a new type of flexible smart material, ionic polymer-metal composite (IPMC) has the advantages of being lightweight and having fast responses, good flexibility, and large deformation ranges. However, IPMC has the disadvantages of a small driving force and short lifespan. Based on this, this paper firstly analyzes the driving mechanism of IPMC. Then, it focuses on the current preparation technology of IPMC from the aspects of electroless plating and mechanical plating. The advantages and disadvantages of various preparation methods are analyzed. Due to the special driving mechanism of IPMC, there is a problem of short non-aqueous working time. Therefore, the modification research of IPMC is reviewed from the aspects of the basement membrane, working medium, and electrode materials. Finally, the current challenges and future development prospects of IPMC are discussed.

## 1. Introduction

Since the concept of smart materials was first proposed in the late 1980s, the research on smart materials has received increasing attention. Smart materials can sense external stimuli and decide how to deal with them properly. The smart material itself is a new type of executable functional material. They can be divided into two categories based on their response to external stimuli. The first category is “property change” materials, where the input of energy results in a chemical or mechanical change in their properties. The second category is “energy exchange” materials that can respond to changes in their internal energy levels, while their material properties remain unchanged [1]. Table 1 lists the smart materials and their defining characteristics in these two product categories.

Among them, electroactive polymers (EAPs) are regarded as a new type of flexible smart material. It can produce large size and shape changes when stimulated by currents, voltages, or electric fields. This is very similar to the phenomenon that biological muscles can change their length under the stimulation of nerve signals; thus, they are called “artificial muscles” [8]. According to the driving mechanism of electroactive polymers, they can be roughly divided into ionic type and electric welding type. Table 2 presents the performance comparison of two typical EAP materials.

Although the electric field type EAP material can produce large deformations under the drive of direct currents, the required excitation electric field is relatively high. On the contrary, ionic EAP uses water or other solutions as a medium to conduct ions, so the excitation voltage of ionic EAP is much lower than that of electronic EAP materials [15]. In addition, compared with the electric field type EAP material, the ionic type EAP material also has the characteristics of low density and being lightweight. Among ionic EAP materials, IPMC has the advantages of fast response, good stability, and large deformation. It has great application potentials in aerospace, biomedicine, bionic machinery, flexible sensors, etc., [16,17,18,19].

The development of IPMC can be traced back to the 1940s. Researchers found that a layer of colloidal silver can be rapidly deposited onto the surface of a membrane to prepare a thin membrane material with conductive function. This is the prototype of IPMC and was first used in ion-exchange membranes for fuel cells in 1959 [20]. Since then, ion exchange membrane technology has attracted great attention. However, the IPMC prepared by this method cannot be used as an electrode because the metal layer is not tightly combined with the polymer surface. With continuous in-depth research, breakthroughs have been made in related technologies. In the 1970s, researchers used the redox method [21] to electroplate precious metals on the surface of the polymer basement membrane, thus solving this problem. Although many researchers have carried out many fruitful studies on IPMC materials in recent years, there are very few mature commercial IPMC material products. Most of the research is still in the laboratory stage, mainly because of the wide variety of preparation processes that still differ in some key steps. This makes the properties of the prepared IPMC materials vary greatly, and it is difficult to meet the needs of practical applications. Due to the small output force, the application of IPMC materials in most occasions is limited [22].

As an emerging smart material, IPMC has similar properties to human muscles and has many advantages unmatched by other materials. The research on IPMC materials has been significantly developed in the past ten years. In this paper, the structural composition and driving mechanism of IPMC materials are firstly analyzed. Then, the preparation technology of IPMC and the modification research work of IPMC in recent years are reviewed in detail, and the performance improvement measures are evaluated. Finally, the development prospect has been provided.

## 2. IPMC Driving Mechanism

**Figure 1:**
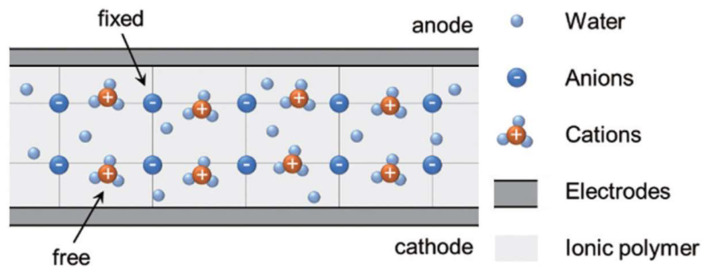
Structure diagram of IPMC [23].

IPMC usually has a sandwich structure, as shown in Figure 1 [23]. It is mainly composed of an ion-exchange membrane in the middle layer and electrodes deposited on both sides of the basement membrane by chemical or physical methods [24]. The ion-exchange membrane acts as a channel for selective ion movement, providing the framework for strain to occur. The ion-exchange membrane for preparing IPMC requires excellent chemical stability and proton conductivity; thus, it is usually a perfluorinated ion-exchange membrane [25].

Commonly used perfluorinated ion exchange membranes include the Nafion membrane, flemion membrane, and alciplex membrane. Compared with the flemion and alciplex membrane, the Nafion membrane has the characteristics of high ionic conductivity, high temperature resistance, and chemical corrosion resistance [26]. Therefore, the Nafion membrane has been widely used to manufacture IPMC [27]. The ion-exchange membrane contains a hydrophobic polymer grid [28], movable ions, and a certain amount of solvent. The internal movable ions provide driving and sensing capabilities. The solvent mainly provides transmission media and channels movable ions, mainly including the most commonly used water, organic solvents, and ionic solutions [29,30,31]. The electrode is a vital part of IPMC, and it has two main functions: One is to act as a carrier of applied voltage and conduct current; the other is to form a capacitor with the intermediate layer that can accommodate a certain amount of charge [32]. The surface resistance of the electrode can directly affect the output performance of IPMC; thus, in the preparation process of IPMC material, in order to reduce the surface’s resistance of the electrode, a thin membrane electrode will be plated on the outside of the initially formed electrode [33].

**Figure 2:**
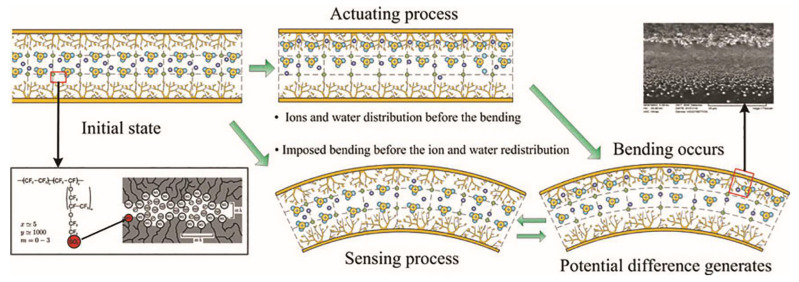
Schematic diagram of IPMC driving mechanism [34].

The driving mechanism of IPMC is shown in Figure 2 [34]. The basic energy exchange mechanism can be described as follows: On the one hand, when a voltage signal is applied to the upper and lower surface electrodes of the IPMC material, a balanced electric field is formed between the metal layers. Subsequently, the movable cations move directionally under the action of an electric field, resulting in the uneven distribution of ion concentration along the thickness’ direction. On the other hand, because the mobile cations combine with water molecules to form hydrated cations, the hydrated cations migrate to the cathode side under the action of an electric field force, and the fixed anions remain on the polymer skeleton. This will cause the contraction of the anode and the expansion of the cathode and macroscopically cause the material to bend towards the anode and deform [35]. During the sensing process, when an external force load is applied to the IPMC material, the material first bends and deforms. At this time, the stress distribution inside the material is uneven, and the ions and solvent molecules are forced to redistribute, resulting in an increase in ions on the expanding side and the shrinking side. The ions are reduced, creating a potential difference between the upper and lower electrode layers [36].

Due to the special driving mechanism of IPMC, water will be electrolyzed during the driving process [37], which greatly limits the life cycle of the material, which poses a serious challenge for researchers to use IPMC in practical applications. In order to solve the problems of the service life and response of IPMC, various researchers have concentrated on the preparation technology of IPMC in order to use it in the actual industry.

## 3. Preparation Technology of IPMC

In order to further improve the output force and dynamic response characteristics of IPMC, as well as improve its durability, a large number of researchers have devoted themselves to the preparation technology of IPMC. At present, in addition to the traditional preparation method of IPMC, its preparation technology can be roughly divided into two categories: electroless plating method and mechanical plating method.

### 3.1. Electroless Plating Preparation Method

The electroless plating method ensures good bonding between the intermediate polymer membrane layer and the electrode layer, which is considered to be a better method for preparing the IPMC sensor than compared to mechanical plating [38]. Electroless plating mainly includes three methods: impregnation–reduction method, reverse electroless plating method, and co-reduction method.

#### 3.1.1. Impregnation-Reduction Method

The most typical preparation method of IPMC in electroless plating is the impregnation–reduction method. The general preparation process is as follows: The polymer membrane is immersed in metal salts to replace the metal cations in the membrane completely. After being taken out, the metal cations are reduced to metal particles with the help of a reducing agent. After repeated experiments, the metal particles accumulate into a certain thickness of metal electrodes [39]. In an early study of chemical deposition by Takenaka et al., a suitable reducing agent was used to reduce the metal ions in the plating solution and deposit them on the surface of the substrate, which was called the “reducing agent penetration method (RP)” [40]. After that, Millet et al. proposed an RP-like impregnation–reduction (IR) method [41], which is to reduce the metal cations infiltrated into the polymer membrane using a reducing agent. Compared with the RP process, the IR preparation method makes the bonding fastness of the electrode layer and the polymer membrane of the IPMC sensor larger, and the electrode is more durable [42]. Palmre et al. [39] prepared the IPMC actuator by the impregnation–reduction method, and its nanothorn electrode can provide a longer life cycle. The actuator was actuated repeatedly for more than 23,000 cycles without a significant drop in displacement amplitude.

**Figure 3:**
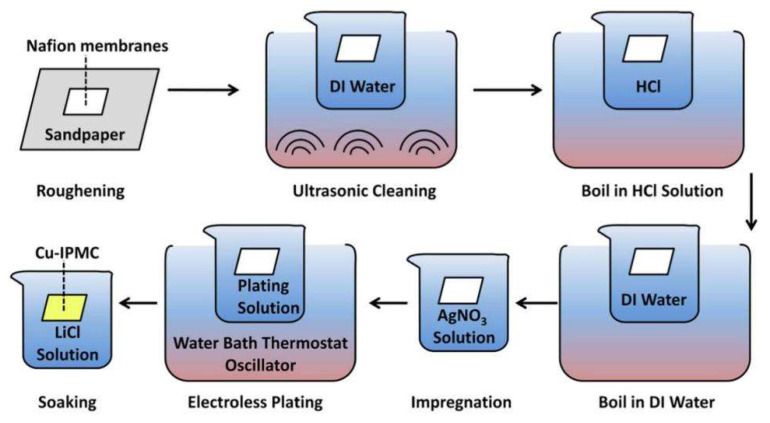
Traditional preparation process of IPMC [43].

The impregnation–reduction method is often used for the preparation of traditional IPMC. The traditional IPMC uses Nafion-117 as the basement membrane material. The preparation process includes four steps: basement membrane pretreatment, ion adsorption, electroless plating, and ion exchange, as shown in Figure 3 [43].

(1) Pretreatment of basement membrane: The ion-exchange polymer basement membrane is one of the important factors determining the driving performance of IPMC. Pretreatment of this membrane can change the chemical stability and proton conductivity of the membrane. The pretreatment of basement membrane mainly includes cutting, roughening, and cleaning. Roughening the membrane can facilitate the deposition of metal particles and is an important method for enhancing the interfacial adhesion between the polymer substrate and the metal electrode [44]. Commonly used roughening methods include sandpaper grinding, sandblasting equipment grinding, plasma treatment, etc. During the grinding process, a large number of hard particles will remain on the surface of the basement membrane. In order to improve the deposition quality of the electrode, the IPMC material needs to be cleaned [45].

(2) Ion adsorption: The purpose of ion adsorption is to provide metal ions for reduction. For example, for Pt-type IPMC, the pretreated basement membrane is soaked in platinum ammonium solution for platinum ion adsorption [46].

(3) Electroless plating: IPMC electrodes are usually prepared by electroless plating. The most commonly used method in the electroless plating process is the impregnation–reduction method. Its realization is based on the principle of the chemical reduction of metals. Firstly, the basement membrane is immersed in a solution containing precious metal ions such as Pt, Au, and Ni. Then, a suitable reducing agent is selected to reduce the metal ions adsorbed on the surface of the Nafion membrane and they are precipitated on both sides of the membrane to form metal electrodes [47].

(4) Ion exchange: Before IPMC is used, it is necessary to replace H^+^ in the Nafion membrane with other cations by ion exchange. The smaller the radius of the metal cation, the better the permeability and the greater the deformation force generated by IPMC. The force generated by the deformation of IPMC containing Li^+^ is the largest, followed by Na^+^, then K^+^, Ca^2+^, Mg^2+^, and Ba^2+^ [48].

In the reduction process of traditional IPMC preparation, since the Nafion membrane is directly placed in the beaker, there is a risk that the Nafion membrane is in contact with the beaker wall or superimposed on each other, resulting in uneven electrode reduction. Moreover, traditional IPMC preparation methods are complicated, time consuming, and expensive, so many scholars are devoted to researching new IPMC preparation technologies.

#### 3.1.2. Reverse Electroless Plating Method

In recent years, Chung et al. [49] developed a new method for preparing IPMC. They plated platinum metal electrode on a Nafion membrane by reverse electroless plating. The specific step is to immerse Nafion into NaBH_4_, and then immerse it in (Pt(NH_3_)_4_)Cl_2_ with different concentrations to trigger the redox reaction. The results show that the prepared IPMC has a longer bending life. It is able to vibrate for 8 h under 5 V at 0.1 Hz drive. By reverse electroless plating, finely dispersed platinum particles can be obtained with a smaller average particle size and a more uniform distribution in the near-boundary region. Compared with the traditional preparation process, the penetration rate of metal particles in the membrane is also higher [50].

#### 3.1.3. Co-Reduction Method

In order to reduce the use of expensive noble metals in the preparation of ionic polymer sensors, Bennett et al. [51] developed a co-reduction method to achieve IPMC for the preparation of noble metal and non-noble metal composite electrodes. The co-reduction method generally refers to the reduction of two different metals onto the same polymer membrane to realize the preparation of IPMC flexible sensors based on composite electrodes. The specific steps are the deposition of an alloy of platinum and copper in a dip-reduction process, followed by using a 50 nm layer of gold to increase the surface conductivity of the electrode. After 6000 cycles with a 3.0 V drive input, the free tip displacement still exceeded 30% of its original value. This shows that the stability of non-precious metal electrodes can be improved by adding a small amount of noble metal as an alloying metal. The metal particle distribution and interfacial area can be controlled by changing the ratio of noble metal and non-noble metal ion concentrations within the membrane.

**Figure 4:**
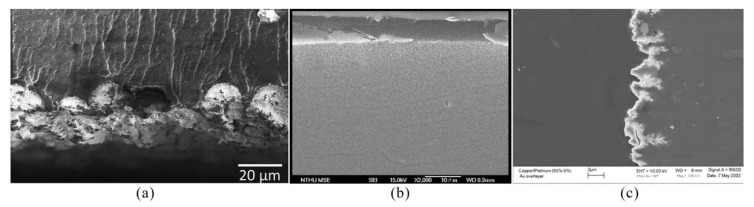
Cross-sectional morphology of IPMC prepared by different electroless plating methods. (**a**) Prepared by impregnation–reduction method [39]. (**b**) Prepared by reverse electroless plating method [49]. (**c**) Prepared by co-reduction method [51].

The cross-sectional morphologies of IPMC prepared by the dipping–reduction method, reverse electroless plating method, and co-reduction method are shown in Figure 4. It can be observed that, in the impregnation–reduction method, the metal cations move to the surface of the membrane and are reduced to the surface of the ion-exchange membrane to form a surface electrode layer; thus, it has a higher surface roughness. The metal particles in the electrode layer prepared by the reverse electroless plating method are evenly distributed, and the surface roughness of the electrode layer is low. However, prepared by the co-reduction method, since the distribution of metal particles can be changed, the interface morphology features are diverse.

### 3.2. Mechanical Plating Preparation Method

Due to the disadvantages of electroless plating, such as being time consuming, having high cost, the batch’s nature, and the inability to mass produce, mechanical plating is more suitable for the preparation of IPMC sensors with non-metallic electrodes. The IPMC electrodes prepared by mechanical plating have good uniformity. However, there is a problem of insufficient bonding fastness between the electrode and the intermediate layer, and the performance is unstable in practical applications [52]. The four commonly used mechanical plating methods are physical vapor deposition, solution casting, hot pressing, and direct assembly.

#### 3.2.1. Physical Vapor Deposition Method

Physical Vapor Deposition (PVD) is a technique for producing hard coatings (thin membranes). According to the difference in the physical mechanism of deposition, physical vapor deposition is generally divided into vacuum evaporation coating technology, sputtering coating, ion coating, and molecular beam epitaxy [53]. The most commonly used method is sputtering, which can be used alone or in combination with other methods such as electroplating and electroless plating [54]. Siripong et al. [55] first deposited 20~30 nm gold on the Nafion membrane by sputtering and then electrodeposited one μm nickel. Sputtering coating provides a simple method for electrode deposition, which greatly shortens the preparation time of IPMC-flexible sensors, and the thickness of the electrode and the roughness of its surface are controllable.

#### 3.2.2. Solution Casting Method

The preparation of flexible IPMC sensors by solution casting mainly includes two aspects: plating conductive layers or increasing the thickness of polymer membranes. Among them, plating conductive layer generally refers to mixing the Nafion solution with a conductive powder, such as carbon, gold, silver, copper, platinum, etc., and then curing at a certain temperature to stabilize the electrode layer [56]. However, this method has strict requirements for the adjustment of variables such as temperature and solution concentration, and there is the problem where it is difficult to repeat. Chung et al. [50] used silver nanopowders to coat the electrode layer in Nafion solution and baked it to ensure the stability of the prepared IPMC membrane. In addition, increasing the thickness of the polymer membrane by casting can enhance the peel strength of the electrode to a certain extent, but from another point of view, the increase in the thickness also increases the difficulty of IPMC deformation. Certainly, the solution casting method provides a feasible means for the shape-variant fabrication of sensors [57].

#### 3.2.3. Hot-Pressing Method

**Figure 5:**
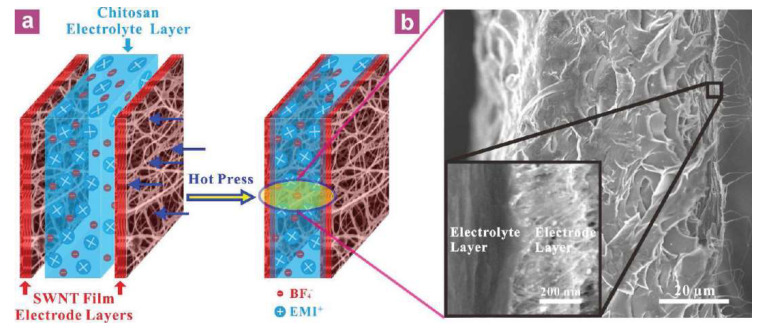
Chitosan actuator based on hot pressing method [59]. (**a**) Schematic diagram of the assembly and bimorph configuration of an actuator. (**b**) Typical SEM cross-sectional image of a bimorph configured actuator.

The hot-pressing method comprises attaching a plurality of relatively thin Nafion membranes and compositing them together with a hot-pressing system so that the polymer membrane prepared for IPMC reaches a specific thickness. Compared with the solution casting method, the hot-pressing method is a simpler process to increase the thickness of the polymer membrane layer, which allows strong repeatability and the easy control of the thickness of the membrane [58]. Li et al. [59] fabricated a novel actuator based on carbon nanotubes and chitosan by using the hot-pressing method. Compared to previous ion-electroactive polymer actuators, multiple aspects of performance have been improved. These include a fast response rate (19 ms), a wider range of available frequencies, and a higher mechanical output power density (244 W/kg), as shown in Figure 5.

#### 3.2.4. Direct Assembly Method

The direct assembly method is mainly used for plating dry membranes. The general operation is to coat the electrode solution on the polymer membrane directly, but at this time, there is a problem that the adhesion between the intermediate polymer membrane layer and the electrode layer is not firm [60]. The membrane is autoclaved to enhance the adhesion between the two. In addition, electrospinning has been used to achieve layer-by-layer self-assembly to prepare conductive network composites. A mat based on co-polymer nanofibers of Nafion and PTFE was developed by Nah et al. through an electrospinning process [61]. Among them, the nanofiber mat is composed of random continuous fibers with a diameter of about 200 nm. Using DAP technology to coat the nano mat can obtain an electrode layer with a thickness of about 25 μm. Compared with the traditional IPMC sensor, the fiber mat allows the response time of the sensor to be improved to a certain extent.

**Figure 6:**
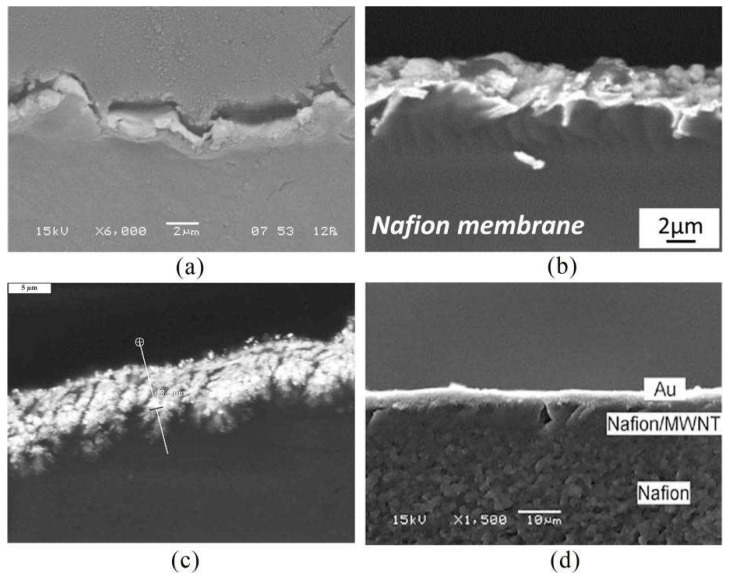
Cross-sectional morphology of IPMC prepared by different physical plating methods. (**a**) Prepared by physical vapor deposition method [55]. (**b**) Prepared by solution casting method [56]. (**c**) Prepared by hot-pressing method [58]. (**d**) Prepared by direct assembly method [60].

Figure 6 shows the cross-sectional morphology of IPMC fabricated by physical vapor deposition, solution casting, hot pressing, and direct assembly. It can be observed that although the density of the deposited membrane is strong in the physical vapor deposition method, due to the intense metal ionization process, more impurity particles will be generated, and the coating surface will be rough. The electrode layer prepared by the solution casting method has uneven membrane formation and poor adhesion of carbon particles to the polymer, which adversely affects the transduction of IPMC. However, by the hot-pressing method, there is no boundary layer between the membranes, and the bonding effect is good. Since the distribution of metal particles can be changed, the interface morphologies are diverse. Fabrication by the direct assembly, assisted by hot pressing, improves adhesion between the electrode layers and membranes, but the adhesion of the conductive surfaces to high surface area electrodes results in performance degradation.

The comparison of electroless plating and mechanical plating preparation technology of IPMC is shown in Table 3. The biggest difference between the electroless plating method and the mechanical plating method is whether there is mutual penetration between the metal particles and the polymer membrane. Penetration can ensure that the IPMC sensor forms an interface layer; that is, the transition layer between the middle polymer membrane layer and the upper and lower surface metal electrodes. Such a transition layer can improve the conduction characteristics of the sensor, which is believed to be due to the increase in double-layer capacitance. Although the electroless plating method is time consuming, complex, and has a relatively high cost, it ensures that there is a good transition layer in the IPMC sensor. Moreover, the electrode layer of IPMC prepared by electroless plating is more durable and has a longer life cycle, which is more energy saving and environmentally friendly than mechanical plating. Although the mechanical plating method has a relatively simple process and short preparation times, the IPMC sensor prepared by this method has no transition layer structure or the transition layer structure is extremely thin.

Although IPMC has the advantages of a low driving voltage, large displacement deformation, rapid response, and being light weight, it has a very broad application prospect as a new type of electric driving material. However, according to the current research results at home and abroad, the existing IPMC still needs to overcome many technical difficulties, such as small output force, short non-water working time, and instability during long-term use [62,63,64]. Therefore, many researchers have studied the modification of IPMC to solve the limitations in its application.

## 4. Study on Modification of IPMC

Although IPMC has many advantages and application prospects, there are still many areas that need to be improved. For example, factors such as strain reduction under DC voltage, low power output, solvent evaporation, and non-standard preparation process limit the application of IPMC. According to the structure of IPMC itself, it can be modified from the following aspects to improve the driving performance of IPMC:

(1) Preparation of basement membranes with diverse structures to meet the diverse needs of IPMC in practical applications.

(2) Optimize the surface electrode of IPMC, reduce the cost of electrode fabrication, and reduce electrode surface cracks.

(3) Optimize the working medium of IPMC, improve its driving stability, and prolong the non-aqueous working time of IPMC.

### 4.1. Study on Modification of IPMC Basement Membrane

The ion-exchange basement membrane has a very important influence on the driving performance of IPMC. The properties of the basement membrane, elastic modulus, water content, migration channels, and other factors will affect the driving performance of IPMC.

**Figure 7:**
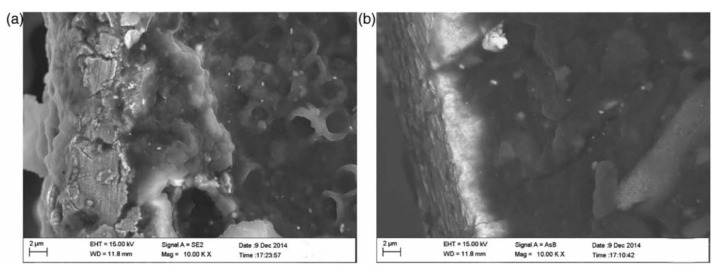
Surface morphology of IPMC composite electrode observed by SEM. (**a**) IPMC electrode surface without MWCNTs. (**b**) IPMC electrode surface with MWCNTs added [65].

A novel ionic polymer–metal composite can be fabricated by adding multi-walled carbon nanotubes (MWCNTs) in Nafion solution [65]. By conducting experiments, it was found that the resistance of the modified MWCNTs/IPMC is much lower than that of the conventional IPMC material. Moreover, under the DC voltage of 4 V, the maximum output force of MWCNTs/IPMC is 3.25 mN, which is 14.84% higher than that of conventional IPMC materials. These significantly improved properties are attributed to the MWCNTs doped into Nafion. The surface morphology of the composite electrode was investigated by scanning electron microscopy, as shown in Figure 7. It was found that the addition of MWCNTs could make the surface coating of the composite electrode smooth and uniform, so the electrode surface resistance is reduced. At the same time, the rigidity of the Nafion membrane is also enhanced to increase its output performance.

Although the Nafion membrane is currently the most widely used fluorine-containing ion exchange membrane, it has the advantages of high chemical stability and mature commercial products. However, in practical applications, fluorine-containing ion-exchange membranes have a high cost, low output force, and retraction problems [66]. Based on the shortage of fluorine-containing ion-exchange membranes, blended composite ion-exchange membranes and novel hydrocarbon backbone ion–polymer membranes were developed to replace fluorine-containing ion-exchange membranes.

The blended composite ion exchange membrane is to improve the actuation performance of the ion exchange membrane by means of polymer blending. One of the high-performance IPMCs based on polystyrene sulfonic acid (PSSA), with PSSA as the hydrophilic proton-conducting functional group, was direct radiation-induced graft-polymerization on polyvinylidene fluoride (PVDF) [67]. Flexible porous membranes were fabricated by a solution casting process. The experimental results show that the PVDF-g-PSSA membrane with the highest engraftment level has the highest average bending strain at 0.1 Hz and 4 V. This is because the porous microstructure of the PVDF-g-PSSA membrane improves the water content and electrical conductivity of the membrane, and the internal nanoscale ionic structure is more conducive to the migration of ions, thereby improving driving performance. Moreover, PSSA is also a water-soluble polyelectrolyte, which can provide free charge carriers to enhance ionic conductivity.

New hydrocarbon main chain ionic polymer membranes include sulfonated polyetheretherketone (speek) and sulfonated poly (styrene-ran-ethylene) (spse); these new ion exchange membranes have the advantages of strong ion exchange capacity, good proton conductivity, low cost, and environmental protection [68].

**Figure 8:**
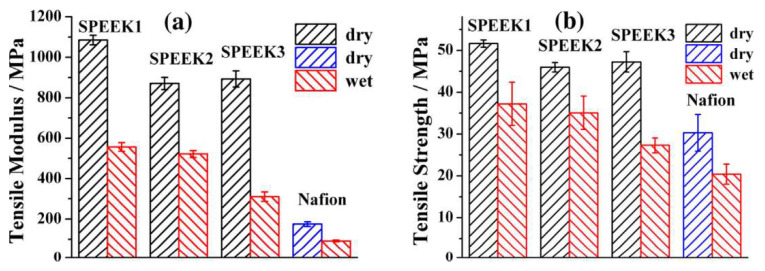
Nafion and SPEEK membranes in dry and wet state. (**a**) Tensile modulus and (**b**) tensile strength [71].

SPEEK membrane has the advantages of excellent chemical resistance, long life, and low cost [69]. The ionic conductivity and mobility of SPEEK are highly dependent on the degree of sulfonation. The reaction conditions such as sulfuric acid concentration, reaction time, and operating temperature can be adjusted by controlling these parameters. SPEEK-IPMC with different sulfonation degrees was prepared for experiments [70]. The experiments showed that the ion exchange capacity, water absorption rate, and ionic conductivity of SPEEK membrane increased with the increase in membrane sulfonation degree, and they were similar to the traditional Nafion membrane. Compared with the Nafion membrane, the high molecular rigidity of SPEEK membrane leads to its higher tensile modulus in both dry and wet states, as shown in Figure 8 [71]. However, the tensile modulus of SPEEK membrane in wet state and intensity decreased with increasing degrees of sulfonation (DS). This is because the absorbed water can act as a plasticizer, reducing the interaction between the SPEEK segment chains.

### 4.2. Study on Modification of Working Medium of IPMC

The bending deformation of IPMC with water as the working medium is caused by the migration of internally hydrated cations; thus, the work of IPMC relies on water molecules in the basement membrane [72]. When the applied electric field is greater than 1.23 V, the water molecules in the IPMC will be electrolyzed, and the long-term operation of the IPMC in the air will cause the water to volatilize to a certain extent. The electrolysis and volatilization of water will result in the gradual reduction in water in the IPMC, which will eventually render the IPMC ineffective [73]. Water as the working medium of IPMC has some disadvantages, so finding a stable working medium is of great significance for the practical application of IPMC.

The results of a series of tests using ethylene glycol as a solvent showed greater resistance to electrolysis and lower stiffness compared to conventional water-based IPMC actuators. This is due to the fact that ethylene glycol is polar, has a viscosity about 16 times that of water at room temperature, has a larger molecular mass, has a greater absorption of the solvent, and has better driving performance and subsequent relaxation in the air [74]. However, compared with ethylene glycol, the IPMC samples with water as solvent drive faster and the samples have higher overall capacitance. Therefore, a large number of studies have used ionic liquids as the working medium of IPMC [75,76,77]. The physical and electrochemical properties of ionic liquids largely depend on the nature and size of their cationic and anionic components. The ionic liquid can solvate and plasticize the Nafion side groups, stimulating the fluorocarbon Nafion host to organize into a microcrystalline structure. The IPMC driving performance is improved by reducing the crystallinity of the IPMC basement membrane and increasing its ionic conductivity [78].

**Figure 9:**
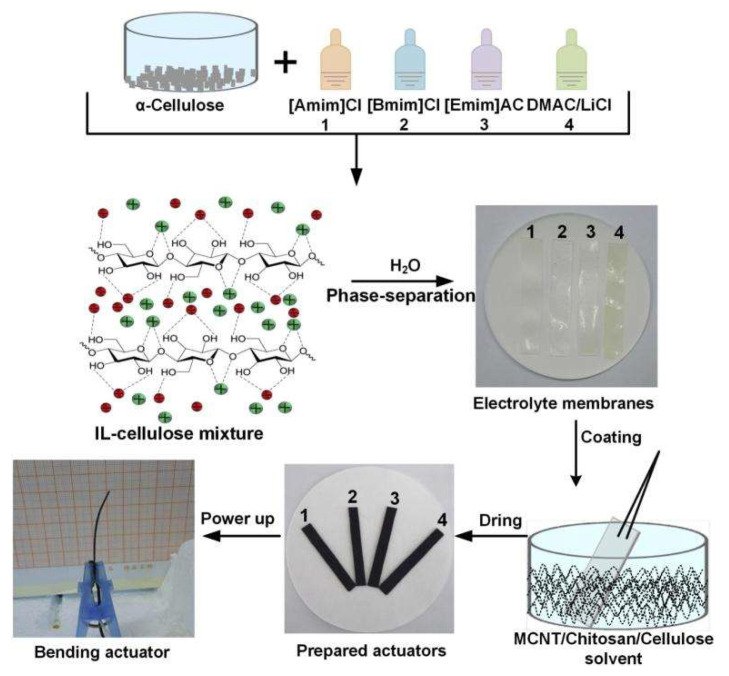
Preparation of biocomposite actuators based on mesoporous renewable cellulose/ionic liquids [79].

Mesoporous regenerated cellulose/ionic liquid (IL-Cel) was used as the ionic liquid medium. A flexible nano biocomposite artificial muscle with a high porosity of 91.31% was prepared, as shown in Figure 9 [79]. The IL-Cel electrolyte membrane prepared by the liquid phase separation method has absolute advantages in flexibility and ion transfer efficiency. The actuator is increased by four times and two times.

**Figure 10:**
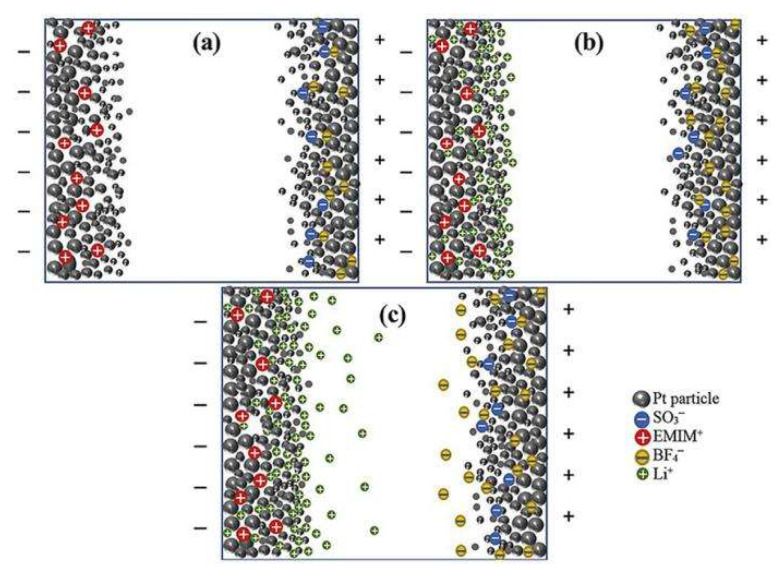
Schematic diagram of IPMC bilayer structures prepared by imidazolium-based ionic liquids with different concentrations. (**a**) Without Li^+^ ions. (**b**) Optimal concentration of Li^+^ ions (0.1 M). (**c**) Higher concentrations of Li^+^ ions (>0.1 M) [48].

IPMC actuators were prepared with different concentrations of imidazolium-based ionic liquids and the actuators were tracked by electromechanical and electrochemical measurement methods, as shown in Figure 10 [48]. The number of free ions in the polymer increases with increasing Li^+^ ion concentration in the IPMC actuator. Because excess ions cannot promote the formation of electric double layers, this can increase the ionic conductivity of the bulk polymer and degrade the capacitive properties of the actuator. The addition of an appropriate concentration of Li^+^ ions to the ion-based system leads to a significant increase in the capacitance, ionic conductance, working life, and displacement rate of the actuator [75].

As the working medium of the driver, the ionic liquid has significantly enhanced the performance stability of the driver. With the development of flexible electrode materials such as graphene, a new type of ionic liquid actuator with chitosan as the polymer substrate and carbon material as the electrode is gradually developed. This type of driver can also show stable output performance in the air.

### 4.3. Study on Electrode Modification of IPMC

Electrodes are very important components in IPMC membranes. Different electrode properties, such as morphology, materials, and electrode thickness, can directly affect the electrical and actuation properties of IPMC. The surface resistance of the electrode directly affects the output force of IPMC [80]. The electrode materials of IPMC can be divided into metal electrode materials and carbon electrode materials. Metal electrode materials are the most used electrode materials at present. Asaka et al. [81] were the first to study the preparation method of Au-type IPMC materials. They obtained Au-type IPMC materials with dendritic infiltration electrodes only by repeated soaking–reduction plating steps. This unique electrode morphology greatly improved the material. However, the organic Au salt used in it has a complex synthesis process and expensive raw materials, so it is rarely used in practice. Therefore, finding an electrode material with excellent braking performance is of great significance for the practical application of IPMC.

**Figure 11:**
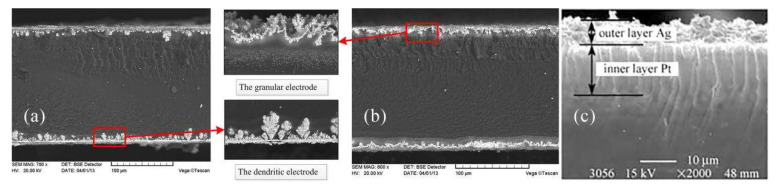
Electrode morphology of IPMC with different metal types. (**a**) Dendritic Au electrode. (**b**) Granular Pd electrode [82]. (**c**) Layered Pt electrode [83].

Due to the hydration requirements of IPMC, attention must be paid to the type of metal used for galvanization. It is critical that metals remain stable under hydration and resist corrosion. At present, the metal electrodes of IPMC are commonly used precious metal materials (Au, Pd, Pt, etc.). IPMCs with different metal–electrode interface properties were prepared by depositing on the surface of the ion-exchange membrane by using an impregnation–reduction process, as shown in Figure 11. From the morphology of the infiltration electrode, Au-type IPMC presents a dendritic electrode, and the infiltration electrode is obvious. Pd-type IPMC is a granular electrode [82], while Pt-type IPMC is a layered electrode [83]. Among the IPMC of the above-mentioned metal electrodes, the Au-type IPMC material has the best performance. This type of electrode has a good binding effect with the basement membrane and has good conductivity and stability. The Pd-type IPMC material has poor performance, and its response speed and displacement are smaller than the other two kinds of IPMC.

Although the metal electrode has high conductivity, the compatibility of metal and polymer is not good. Inevitably, the metal electrode and the ion exchange membrane will flake off during repeated use, which affects the actuation performance of the IPMC membrane [84]. To develop high-performance IPMCs, highly conductive carbon materials are used as electrodes for IPMCs. Carbon nanotubes and graphene show strong application prospects due to their unique physical properties and structural advantages, such as large surface area, high electrical conductivity, and high mechanical strength [85].

**Figure 12:**
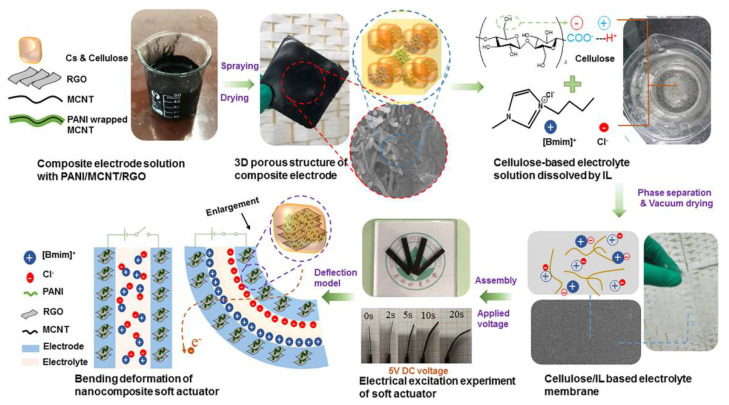
Preparation of renewable biocompatible nanocomposite soft actuator based on three-dimensional porous electrode [86].

First, highly conductive polyaniline doped with reduced graphene oxide/carbon nanosheets and a natural biopolymer cellulose-based electrolyte constitute a 3D porous electrode. Subsequently, a renewable biocompatible nanocomposite soft actuator was prepared, as shown in Figure 12 [86]. The actuator enables large deformations, high-performance flexibility, good breathability, and highly durable muscle performance.

The modification studies of IPMC from three aspects of the basement membrane, working medium, and electrode are shown in Table 4. It can be observed that the modification from the basement membrane can improve the driving performance by increasing the rigidity of the IPMC. Modification from the working medium can improve the stability and working life of IPMC. Modification from the electrode can improve the conductivity of IPMC. Modification from the above three aspects has its own advantages and disadvantages, so scholars will conduct modification research on two or three aspects to improve the driving performance of IPMC.

## 5. Conclusions

IPMC materials have many excellent properties, such as large deformation, fast response time, and low driving voltage, which allow them to continue to be one of the research hotspots in smart materials. Because of its unique mechanical and electrical properties, it can be well used in drivers, sensors, etc. However, it is precisely because of the special driving mechanism of IPMC that water will be electrolyzed during the driving process, resulting in many problems of IPMC, such as small output force, short non-water working time, etc. In order to solve these problems and improve the driving performance of IPMC, researchers have conducted a lot of research work, starting from the preparation technology and modification of IPMC.

In terms of preparation technology, the IPMC prepared by electroless plating can form a transition layer well and improve the driving performance. Although the IPMC electrode prepared by mechanical plating has good uniformity, the bonding fastness between the electrode and the intermediate layer is not sufficient, which will result in the instability of IPMC. Therefore, IPMC prepared by electroless plating, even if it is expensive, has more stable performance than IPMC prepared by mechanical plating; thus, it is more widely used than mechanical plating. In terms of modification research, researchers have carried out modification research from three aspects: IPMC basement membrane, working medium, and electrode material. By modifying different aspects, the different properties of IPMC are improved, such as improving the rigidity of the basement membrane, the conductivity of the electrode, and the stability of the IPMC.

A lot of work is still needed for research before IPMC is widely used. Since IPMC is generally prepared by electroless plating, it faces the challenge of high cost and time-consuming preparation. It is necessary to develop an economical and time-saving preparation technology. Most of the current work focuses on driving IPMC, and there are few research studies on sensing IPMC. Therefore, using the driving characteristics of IPMC, it is widely used in bionic robots. If the problem of electrode delamination and cracking during the use of IPMC can be overcome, and the system integration method of IPMC and flexible products can be explored, IPMC can be used as a wearable sensor. It will have broad application prospects in the fields of medical monitoring and sports fitness.

## Figures and Tables

**Table 1:** Smart material classification.

Type	Material	Stimulant	Result Impact	Ref.
Property changes	Thermochromic	Thermal energy	Spectral reflectance	[2]
	Magnetorheological	Magnetic field	Viscosity	[3]
	Shape memory	Thermal energy	Crystal phase transition	[4]
Energy exchanges	Photovoltaic power generation	Radiant energy	Electric current	[5]
	Electroactive polymer	Electric field	Stress	[6]
	Thermoelectric	Electric current	Temperature difference	[7]

**Table 2:** Performance comparison of different types of EAP materials.

Type	Category	Strain Rate (%)	Stress (MPa)	Response Time	Stiffness (MPa)	Density (g/cm^3^)	Drive Voltage	Ref.
Ionic type	IPMC	>20	10~30	ms~s	70~300	1~3	1~5 V	[9]
	Conductive polymers	0.1	5	ms~s	>10^3^	1.48	<10 V	[10]
	Ionic gel	40	-	min	0.1	1.1	0 V/mm	[11]
Electric field type	Dielectric elastomer	300	0.2	ms	0.5	1.5	144 V/μm	[12]
	Ferroelectric polymer	2~10	45	ms	1~10^3^	1.78	200 V/μm	[13]
	Electrostrictive elastomer	1.7	65	ms	600	7.5	12 V/μm	[14]

**Table 3:** Comparison of IPMC preparation technology.

Category	Preparation Method	Advantage	Disadvantage	Ref.
Electroless plating	Impregnation–reduction method	Forms durable metal electrode layers	Time consuming and costly	[41]
	Reverse electroless plating	More uniform metal particle distribution	The process is complicated	[49]
	Co-reduction method	Non metallic materials can be used	Time consuming and costly	[51]
Mechanical plating	Physical vapor deposition	Time saving and large area preparation	Poor bond fastness	[55]
	Solution casting method	Diversified shapes	Need to be used with other methods	[56]
	Hot-pressing method	The thickness of the membrane is controllable	Poor flexibility	[58]
	Direct assembly method	Easy to operate	Poor interface bonding	[60]

**Table 4:** Study on modification of IPMC.

Modification Aspect		Advantage	Disadvantage	Ref.
Modification of basement membrane	MWCNTs/IPMC	The surface coating is smooth and uniform, and the rigidity of the basement membrane is enhanced	There are problems with low output force and retraction	[65]
	Blended composite ion exchange membrane	Conducive to ion migration and improve driving performance	Higher cost	[67]
	Hydrocarbon backbone ion polymer membrane	Good proton conductivity, low cost and environmental protection	Time consuming to prepare polymeric membranes	[71]
Modification of the working medium	Organic solvents	Improve the stability of the basement membrane	Slow driving speed	[74]
	Ionic liquid	Improve drive performance and operating life	Higher cost	[79]
Modification of electrodes	Rare metals	Good conductivity and stability	Easy to fall off after repeated use	[82]
	Carbon material	High performance, high conductivity	Higher cost	[86]

## Data Availability

Not applicable.
